# “What if the patient has a severe reaction, and it is my fault?” A qualitative study exploring factors for sustainable implementation of penicillin allergy delabelling

**DOI:** 10.1186/s13756-024-01456-8

**Published:** 2024-09-02

**Authors:** Marie Bjørbak Alnæs, Brita Skodvin, Jan Anker Jahnsen, Grete Kalleklev Velure, Oddvar Oppegaard, Bård Reiakvam Kittang, Torgeir Storaas, Margrethe Aase Schaufel

**Affiliations:** 1 Section of Clinical Allergy, Department of Occupational Medicine, Haukeland University Hospital, Bergen, 5020 Norway; 2 Department of Clinical Medicine, University of Bergen, Bergen, 5021 Norway; 3 The Norwegian Advisory Unit for Antibiotic use in Hospitals, 5020 Bergen, Norway; 4 Regional Medicines Information and Pharmacovigilance Centre (RELIS Vest), University Hospital, Haukeland, Bergen, Norway; 5 Department of Medicine, Haukeland University Hospital, Bergen, 5020 Norway; 6 Haraldsplass Deaconess Hospital, Bergen, 5009 Norway; 7 Department of Nursing Home Medicine, Fyllingsdalen, 5145 Norway; 8 Department of Thoracic Medicine, Haukeland University Hospital, Bergen, 5020 Norway

**Keywords:** Penicillin allergy delabelling, Nurse, Doctor, Facilitators, Barriers, Focus group interview, Qualitative research, Implementation

## Abstract

**Background:**

Penicillin allergy delabelling (PAD), the process of evaluating penicillin allergy labels, is a key target in antibiotic stewardship, but uptake of the procedure outside clinical studies is limited. We aimed to explore factors that need to be addressed to sustainably implement a clinical pathway for PAD.

**Methods:**

We conducted a qualitative study based on semi-structured interviews with focus groups consisting of a purposive sample of twenty-five nurses and physicians working in four different hospitals in Western Norway. Systematic text condensation was applied for analysis.

**Results:**

Psychological safety was reported as crucial for clinicians to perform PAD. A narrative of uncertainty and anticipated negative outcomes were negatively associated with PAD performance. Education, guidelines, and colleague- and leadership support could together create psychological safety and empower health personnel to perform PAD. Key factors for sustainable implementation of PAD were facilitating the informant’s profound motivation for providing optimal health care and for reducing antimicrobial resistance. Informants were motivated by the prospect of a simplified PAD procedure. We identified three main needs for implementation of PAD: (1) creating psychological safety; (2) utilising clinicians’ inherent motivation and (3) optimal organisational structures.

**Conclusion:**

A planned implementation of PAD must acknowledge clinicians’ need for psychological safety and aid reassurance through training, leadership, and guidelines. To implement PAD as an everyday practice it must be minimally disruptive and provide a contextually adaptive logistic chain. Also, the clinician’s motivation for providing the best possible healthcare should be utilised to aid implementation. The results of this study will aid sustainable implementation of PAD in Norway.

**Ethics:**

The study was approved by the Western Norway Regional Committee for Medical Research Ethics (Study No:199210).

**Supplementary Information:**

The online version contains supplementary material available at 10.1186/s13756-024-01456-8.

## Background

Penicillin allergy delabelling (PAD) is the process of evaluating penicillin drug allergy warnings and removing false penicillin drug allergy warnings. Traditionally this has been done in allergy departments, where patients had their penicillin allergy history taken, underwent skin testing and serum measurements of specific IgE towards penicillins before penicillin re-exposure. Over the last years methods for risk-stratification based PAD outside allergy clinics have been developed. In these, patients have their penicillin allergy history taken by physicians not specialising in allergology. Adhering to preformed criteria patients are risk-stratified into categories correlating with their risk of true penicillin allergy. Patients with a low risk of true penicillin allergy are then directly re-exposed to penicillins in a controlled oral penicillin challenge, whereas high-risk patients are still referred to an allergy department for further testing. PAD is one of the cornerstones in antibiotic stewardship and is recognised as an important measure for preventing antibiotic resistance [1, 2]. Nine out of ten patients claiming to be penicillin allergic have been found to tolerate penicillin when subjected to allergologic testing, suggesting an unmet need for systematic evaluation of patients with alleged antimicrobial allergies [3]. PAD guidelines have been developed in many countries, but sustainable dissemination and implementation of these outside allergy units is rare [4–6].

Previous analyses of PAD programs have revealed the need for multifaceted efforts to succeed [7]. Moreover, the importance of qualitative research concerning PAD has been demonstrated, and the clinician’s perspective appears to be crucial for the sustainable development and implementation of PAD programs, for the benefit of patients and health care systems [8]. Nevertheless, knowledge about clinicians need to perform PAD is scarce, and only two interview studies from Great Britain and the United States of America have addressed this issue in-depth [9, 10]. In these studies, PAD was regarded as a complex task and not a priority during acute patient presentation. Also, time restraints, a lack of securely communicating penicillin allergy labels and a fear of inducing an allergic reaction were barriers for performing PAD. As the education of health personnel in PAD-, legislation- and health systems are subject to contextual differences, studies from various health care systems are needed [8]. We have earlier developed and validated a PAD in Norwegian [5], but before implementing PAD on a broader scale we needed knowledge to aid implementation. Hence, we performed an interview study with nurses and physicians working in Western-Norway Health Region (WNHR) hospitals to explore how PAD is perceived and identify needs to be met for the sustainable implementation of a clinical pathway for PAD in Norway.

## Methods

### Study aims

This study explores how nurses and physicians in the WNHR manage declared penicillin allergy and what needs must be met to sustainably implement a clinical pathway for PAD in Norway.

### Study setting

We performed a qualitative study consisting of semi-structured focus group interviews with physicians and nurses working in hospitals of all service levels (university-, regional- and local hospitals) in the WNHR. The hospitals have a total catchment area of 1,14 million patients [11]. The interviews were performed between September and December 2023. The Norwegian health system is predominantly state funded and governed. Norway has a national action plan [12] for fighting antimicrobial resistance and antibiotic stewardship is mandatory in Hospitals. In Norway penicillins are the first choice of antibiotic treatment in most settings [13].

### Ethics

The study was approved by the Western Norway Regional Committee for Medical Research Ethics (study No: 199210) and all participants provided informed written consent both for participation and for publication of the data. The study results are reported in line with the Consolidated Criteria for Reporting Qualitative Research (Supplement 4) [14].

### Participants

The informants were recruited for interviews by the local clinical leader and then invited to participate via email. The local clinical leaders were physicians and nurses meant to lead the implementation of penicillin delabelling in their departments later on. They were well known to the informants as established leaders of the local medical teams. Two of the local clinical leaders also co-supervise the first author in her PhD. The informants all knew that the study was a part of preparing for implementing PAD in their departments and that the study is part of the interviewers PhD project. The interviewers professional background and employment in WNHR was known to the informants. The informants had varied professional backgrounds, including dermatology, pulmonology, otorhinolaryngology, internal medicine, emergency medicine, rheumatology, and infectious diseases. The informants’ level of experience in PAD differed. Three informants had broad experience in PAD and worked in an outpatient allergy clinic, however, none of these informants had experience in penicillin delabelling without prior skin and serum testing. The other informants worked in internal medicine departments, emergency medicine departments and infectious disease departments. Of these, eight stated no previous experience with PAD, whereas the others declared that they had some experience with PAD but lacked formal training in using the procedure. The demographics of the informants are presented in Table 1. Participation was voluntary, and informed written consent was obtained before commencing the interviews. The informants were selected to represent all levels of experience and types of hospitals in the WNHR. The groups were recruited as purposeful samples, aiming at diversity in experience, gender, professional background, and age.

**Table 1:** Demographics

Distribution of informants	Female	Male	Total
Total number of informants	15	10	25
**Nurses**	11	1	12
Nurses with ≤ 5 years of practice	3	0	3
Nurses with ≥ 5 years of practice	8	1	3
**Physicians**	4	9	13
Residents	1	2	3
Board certified specialist doctors	3	7	10

### Theoretical perspectives

“A Checklist for identifying determinants of practice” was chosen as a framework informing the interview guide [15]. According to this framework, tailored implementation interventions are strategies designed to improve health care. Examining the challenges that may occur when implementing such interventions will aid in better and more targeted implementation. This can increase uptake of interventions. The framework constituted a basis for assessing and prioritizing determinants for exploration in the study and aided the development of interview questions adapted to our context. In the interview guide (Supplement 1), the chosen determinants are listed above each question. We purposely kept questions open to encourage the informants to report their unsolicited thoughts, experiences and needs in the context of implementing penicillin allergy delabelling in their department.

### Data collection

Three of the authors (MBA, BS, and MAS) developed the interview guide (*Supplement*1). MBA is a Norwegian female physician working at an Allergy department in the WNHR, and has a special focus on drug allergies, particularly PAD. MBA has no earlier experience in qualitative studies and the work is part of her PhD project. The co-authors BS and MAS, who co-created the interview guides, are also Norwegian female physicians, but have broad experience in qualitative research. We developed a guide for focus group interviews as we deemed it the most suitable way of detecting the necessary knowledge, as PAD is a complex intervention dependent on interdisciplinary cooperation. The interview guide was refined after the second interview to optimise the questions and adjust for the allocated timeframe. MBA performed the interviews using a semi structured interview guide (Supplement 1). The first interview was conducted with only one physician due to acute staffing shortage in the clinic, one interview was an online meeting with Teams© (Microsoft) due to organisational challenges. The other interviews took place in meeting rooms at the participants workplaces and lasted 60 min. Only the interviewer and the informants were present during the interviews. The focus groups consisted of 3–5 informants. One group consisted of only nurses, one of only physicians and the remaining groups had both nurses and physicians as informants. Table 2 (Supplement 2) describes the distribution of informants in the focus groups.

### Analysis

Transcripts were analysed with systematic text condensation, a pragmatic method for cross-case, thematic analysis [16]. Systematic text condensation analysis contains four steps: (1) Obtaining an overall impression by reading the complete material (2) Identifying units of meaning containing the participants’ attitudes and opinions towards performing PAD and coding these units (3) Condensing and abstracting meaning from the coded groups. (4) Summarising together with creating concepts and descriptions from the condensates.

MBA, BS, MAS, GVK, and JAJ participated in the analysis. Initial overview and coding were done by each author alone, further analysis and coding was done as a team. When there were different views on the coding, agreement was reached through discussion. The analysis was performed as a stepwise-, cross-sectional-and iterative analysis in a hybrid approach, combining inductive- and deductive analysis and adhering to recommendations for the application of systematic text condensation. An audit trail was kept throughout the study and all choices and changes (such as moving from a planned in-depth interview study) were made traceable here. The audit trail consists of text files with meeting minutes, notes from creating and executing the study and field notes from the interviews. Also, the notes made throughout the analysis are part of the audit trail. The analysis was documented in Word (Microsoft) files. A secure “Teams” (Microsoft) room and in person meetings were used for the analysis, and the participating researchers had access to the running files and all the decisions made throughout the analysis.

### Language and translation

The interview guide was constructed in Norwegian, and the interviews and analyses performed in Norwegian. The overview of the analysis process (Supplement 3) and informant quotes were translated by the authors. The semi-structured interview guide (Supplement 1) was translated initially by professional translators (Translated©) and then refined by the authors. All translated text was translated back and forth by the authors to ensure accuracy.

## Results

**Fig. 1:**
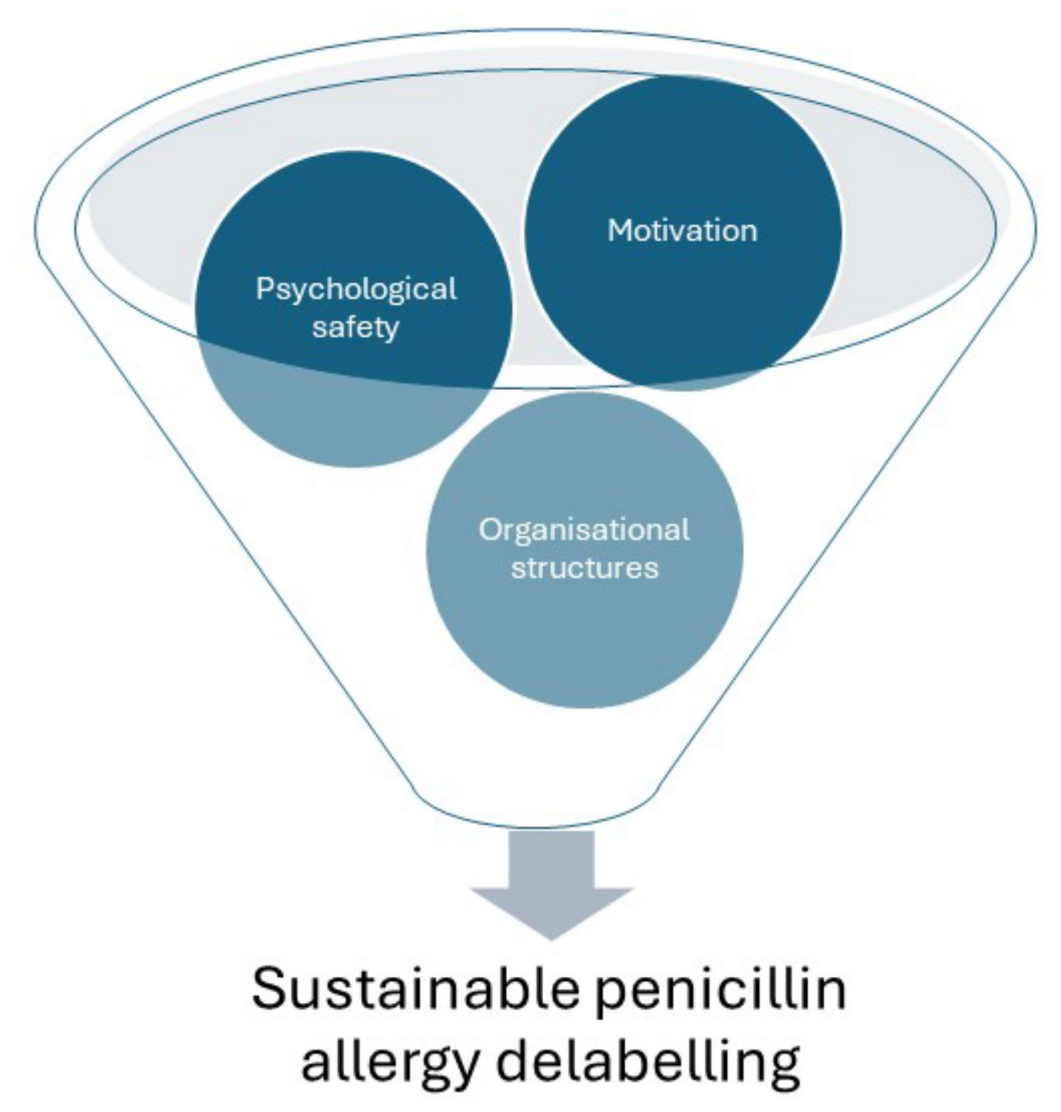
Factors needed for the sustainable implementation of penicillin allergy delabelling

We identified three main needs for implementing PAD: (1) creating psychological safety; (2) utilising clinicians’ inherent motivation to aid implementation; and (3) providing optimal organisational structures. Below, we elaborate our findings illustrated by quotes from the transcripts. Figure 1 demonstrates that all of these components are needed for clinicians to perform PAD.

### Creating psychological safety

#### Clinicians’ anxiety when performing PAD must be addressed and decreased

The informants highlighted psychological safety as crucial for performing PAD. To experience that PAD is supported throughout both their scientific-, and medical communities, and in the workplace was essential, as several physicians expressed a profound sense of uncertainty and anxiety towards PAD. They felt a knowledge gap concerning PAD, and physicians associated PAD with an inherent risk. This was most prominent in physicians who had never participated in penicillin delabelling. Fear concerning outcomes for patients, with a perceived high risk of reactions to provocation testing, and fear concerning personal and professional consequences if the patient reacted to the penicillin challenge were central aspects. The most feared adverse outcome was anaphylaxis and death due to anaphylaxis. One of the doctors described it as follows:

“I feel that we are unsure, lack experience and need to be empowered. We need support both from guidelines, from leaders and colleagues in addition to more knowledge of the field. I am too unsure of the method we use today, and then I do not do it. You know, we are trained to respect drug allergy labels. Often, we will discuss penicillin delabelling, and are quite convinced that the patient has no penicillin allergy, but then we decide not to perform penicillin delabelling because you know, there could be a very steep fall when overruling a drug allergy warning. Both for the patient and for the doctor. What if the patient has a severe reaction, and it is my fault.” Doctor #5.

The high level of fright towards adverse reactions, and anaphylaxis in particular, when performing PAD contradicts the lived experience from the physicians that had performed PAD, as none of them had ever experienced any severe adverse events when delabelling patients.

“Well, the experience over the 13 years I have done delabelling is that there has never been a severe reaction, no anaphylaxis, and I have been quite liberal in my delabelling.” Doctor #7.

#### Clinicians need empowerment and support to perform PAD

The informants sought empowerment and communicated a need for more knowledge, training, and experience in PAD. They craved information and partly used the interview setting to educate themselves, asking the interviewer several questions about PAD throughout the sessions. They wanted to be able to do PAD and to do it right; to be educated and participate in performing PAD provided increased psychological safety. They were aware that they need to change today’s practice and suggested both lectures and simulation training to make them feel safe to perform PAD. They emphasised that they learn most from each other, as apprentices learn from their masters. The informants asked about opportunities for training in PAD both as scenario training, including managing adverse events with an emphasis on anaphylaxis, and the opportunity to visit clinics performing PAD today to learn the method hands on.

The participants said that clear guidelines are crucial. The knowledge that government- and medical society led support is available were mentioned as key factors towards establishing psychological safety enabling them to perform PAD. Concerns voiced towards the current state of guidelines concerning PAD were that they are hard to find, outdated and partly contradicted each other. Readily available guidelines that coherently communicate PAD and focus on the practical everyday needs concerning the act of delabelling patients were missed.

The nurses all relied on the doctor’s decision concerning delabelling declared penicillin allergy. They said that they do whatever the doctor decides. The nurses were also less nervous towards adverse reactions to the provocation testing as most of them stated that they had performed PAD earlier with skin testing and administered intravenous antibiotic test doses on rare occasions.

There was an overall call for trust and need for support to perform PAD. The clinicians wanted leaders that installed a trust in them that performing PAD was safe and supported. The informants explained that they would only do PAD if they had a clinician they trusted by their side. The feeling of safety and trust within the team was reported as a major facilitator towards implementing PAD. Physicians and nurses reported that they would need support from their peers to perform PAD regularly.

“I want it to be a doctor I trust, one I am sure will appear quickly when paged, and that we as a team decide to perform PAD. I must know that we are prepared if a reaction occurs.” Nurse #2.

“That I know the procedure, the leadership endorses the procedure and that the other doctors agree, and I am working with a nurse that agrees to the procedure, then I would trust myself to do PAD, then it would feel safe.” Doctor #3.

### Utilising clinicians’ inherent motivation to aid implementation

#### Clinicians want to provide the best possible healthcare

The clinicians’ main motivation and main facilitator for aiding in the sustainable implementation of PAD was the prospect of optimising patient care and reducing the use of broad-spectrum antibiotics. Providing the best clinical care was the main interest of clinicians and the fact that current practice was not ideal was perceived as shameful.

“I want to provide the best patient treatment possible. I also want to reduce antimicrobial resistance. And our current method is outdated and rarely performed, we simply must change. I see it as a win-win situation. I mean concerning antibiotic resistance and the patient. It is easier for us to perform delabelling this way. We heal patients from their declared penicillin allergy and can give them the most efficient treatment. Not being able to receive penicillin is quite severe, a disease on its own come to think about it, and changing that, well that is the reward.” Nurse #8.

#### Clinicians want to reduce antimicrobial resistance and negative impacts of penicillin allergy labelling

The knowledge of the incremental harm of false penicillin allergy labels was high concerning antimicrobial resistance. Knowledge concerning the other negative impacts of penicillin allergy labels such as increased morbidity and mortality of the individual patient, increased cost of treatment and longer hospital stays was low. The increased awareness of these topics throughout the interviews, and the possible benefits of PAD, were immediately mentioned as important motivations for them to change their practices and attitudes towards PAD. This knowledge was also mentioned to motivate them in discouraging the common practice of simply choosing another antibiotic when the patient declares penicillin allergy, as an easy way out, as the harm in this practice dawned on them.

“I have been annoyed by all the patients declaring themselves penicillin allergic for uncertain reasons, and then I must prescribe them a broad-spectrum antibiotic. But I was unaware how bad it is overall it is for the patient to be labelled penicillin allergic for no reason, and that our procedures today are so uncertain and outdated, that feels very unsettling.” Doctor #3.

#### Clinicians are motivated by the prospect of a simplified clinical procedure

The nurses were particularly motivated by the prospect of a simplified clinical procedure, as they perform the test procedure and have the highest workload in PAD. Many of them reported having performed skin-testing and provocation-tests for penicillin allergy earlier and reported that the new method represented time and resources saved on their behalf. This was reported to be a strong motivation. It also demonstrates a dissatisfaction with the current systems.

“I like that it seems easier for us nurses to perform. I believe it is a lot less stressful to just administer a tablet than to perform today’s skin testing, with dilution series of the medication and sorting syringes, it’s quicker too.” Nurse #4.

### Providing optimal organisational structures

#### Clinicians need a seamless workflow adapted to their working context

The informants emphasised that an optimal organisational structure was the main prerequisite implementing a clinical pathway for PAD in everyday hospital practice. They called for a preformed logistic chain and for the method to be readily available to be able to perform PAD. This was due to their experienced lack of time in everyday practice as an obstacle for PAD, together with an undefined responsibility of when and where to perform PAD. Streamlining the process would increase uptake and make PAD feasible.

“If we are to do this on an everyday basis, then we need a system for it in the patient journal, so that it is easy to remember, and quick to perform. If we must start searching for a form or something, it will not happen. And we need to know where and when it is expected for us to do this.” Doctor #1.

The clinicians wanted a clinical pathway for PAD to be available across all platforms and media used in their everyday practice. As one size does not fit all, PADs should be available in both paper forms, mobile phone apps and electronic versions in patients’ health care charts. Nudging, described as automated electronic reminders in the electronic health records was met ambivalently as they were often perceived to obstruct the workflow especially by the physicians, even though their function as clinical reminders was acknowledged as useful.

“I experience that we get more and more patients, more and more tasks to perform, and less and less time for each patient, so it must be readily available when I need it, in the systems we use already. So that we do not forget to do it, and minimal extra hassle occurs. It needs to become sort of second nature for it to be a sustained method.” Doctor #10.

Both nurses and physicians called for better tools for delabelling. They defined tools both as preformatted work sheets describing the process of PAD and software for PAD guidance integrated in the local electronic patient records. The tools were mentioned as necessary everyday prerequisites for aiding the delabelling process on a case-by-case basis.

“l would like it to be like a cooking recipe, and with a preformed scoring system that made the decision to perform delabelling clear and the procedure to do so easy. The guidelines ought to be as identical as possible, in the hospital, in the national antibiotic guidelines and elsewhere.” Doctor #2.

#### Clinicians need appointed clinicians to lead implementation

All the informants highlighted the importance of clinical teams and “lighthouses,” meaning clinicians especially appointed for both educating and reminding other colleagues to perform PAD. They believed that if the clinical pathway for PAD was embedded into their everyday routine with minimal disruption it would pave the way for sustainable implementation of PAD. Additionally, there was a call for well-functioning interdisciplinary teams, as PAD is a complex intervention necessitating cooperation between physicians and nurses. The nurses emphasised their position as gatekeepers and reminders of best practices within the medical team. Physicians often rotate between wards during their time on call, as opposed to nurses. The nurses reported that they could use this position to remind the team to perform PAD and aid the sustainability of PAD programs. The informants pinpointed that the everyday working order of each profession and department must be considered when creating and implementing the PAD workflow.

“I believe most of us nurses would ask the doctors about penicillin delabelling. Especially during prerounds preparation. But it is a team effort, and you need to have someone to trailblaze it. We have antibiotics teams that could take charge and pull the other with them.” Nurse #9.

## Discussion

To the best of our knowledge this is the first qualitative interview study providing an in-depth understanding of the perceived needs of clinicians when implementing PAD in Scandinavia. The informants described their knowledge and motivation for PAD, but also their anxiety and shortcomings concerning performing PAD today. The development of sustainable programs and guidelines [17] paves the way for sustainable implementation, but true sustainable development only occurs when new knowledge and guidelines are implemented in everyday practice [18]. Our study provides added information on how to make a clinical pathway for PAD fit to practice and identifies several targets for efforts that will help improve the implementation of PAD in Norway.

### Psychological safety

Our main finding was that clinicians need psychological safety to perform PAD. The informants were highly motivated to perform PAD but were at the same time unsure and anxious to do so. Anxiety is multifaceted, and both the anticipated fears concerning the patient, concerning themselves, and the fear of professional consequences must be addressed. Their glimpse of potential danger, in particular inducing anaphylaxis in a patient, overshadowed their knowledge of benefits and colleagues’ reports of successful delabelling experiences. The level of anxiety was also surprisingly high considering the large body of international research on PAD that deems it safe and provides both guidelines and tools for perform delabelling [19, 20]. Psychological safety has been mentioned as a barrier for performing PAD in a recent study from the USA [10] but was reported more profoundly by our informants than in this previous study. The Norwegian health care system is less hierarchical than most health care systems outside Scandinavia. The level of trust in society and the health system is among the highest in Europe [21]. Adding to this, an increased awareness of clinicians’ anxiety and errors in Norway in recent years has been observed [22]. These social factors probably influenced our results and made it easier for our informants to openly address their professional anxiety concerning penicillin allergy delabelling.

Previous studies have revealed that clinicians report PAD to be a complex issue, and they perceive it challenging to examine and communicate penicillin allergy labels. They reported that time to perform PAD is lacking and that they are unsure about who is meant to perform PAD [9, 23, 24]. We believe that a lack of psychological safety might be a component of several of the other issues raised, as the main fear our informants reported when asked to perform PAD was that their patient might experience an adverse reaction to penicillin after being delabelled. This concern has also previously been reported as the main reason why general practitioners and hospital clinicians do not amend their patient records or prescribe patients penicillin, even after the patient has undergone negative penicillin provocation tests [25–28]. In addition, our informants deemed most of the obstacles reported in earlier studies (such as time restraints, practical logistics in performing PAD and reporting results) manageable if they just felt safe to perform PAD. Furthermore, our informants confirmed that they needed the trust of their organisation and the trust within the team across professions, to feel safe performing PAD. One might say that they needed this external trust to trust themselves, and trust-endorsed processes have been mentioned as necessary in PAD in an earlier study [9]. The level of trust in interprofessional health-care teams has been examined in several health care settings and trust in the whole team is deemed a significant enhancer of performance and necessary for the safe delivery of health care [29, 30]. Ensuring that clinicians feel that it is psychologically safe to perform PAD will likely ease other barriers in PAD, as psychologically safe clinicians have been shown to be more effective and safer in delivering healthcare, demonstrating improved organisational learning at the same time [31], all key factors to sustainable implementation of PAD.

### A need for guidelines and “mindlines”

All informants mentioned that guidelines supporting PAD were crucial if they should perform PAD on an everyday basis, and all reported adhering to the national guidelines concerning PAD. However, none of the clinicians had noticed that the national guideline for PAD had been removed from the internet more than a year before the interviews commenced, as it was deemed outdated by the Norwegian Directorate of Health. This demonstrates that guidelines are not necessarily used on a regular basis; rather the mere knowledge that they exist and support their practice, empowers clinicians. Moreover, the embodied and self-perceived knowledge of a topic determines their clinical choices. The word “mindlines” has been introduced to describe the internalised knowledge, ethics and clinical practice each clinician and team possess and act upon on a day-to-day basis [32]. We found that the “mindline”-led practice of each team was decisive for how motivated clinicians were to perform PAD. Teams with positive experience in PAD, knowledge of the harms of erroneous penicillin allergy labels and knowledge that their practices concerning PAD today were inferior, were more inclined to implement PAD. This approach adheres to knowledge from organisational theory and implementation science, beyond the particularities of PAD [33, 34].

### Motivation

The informants in the focus groups were aware that penicillin allergy labels increase the risk of multiresistant bacterial infections. This finding is in line with earlier studies [10, 26, 35]. Their knowledge gaps regarding the other known negative impacts of penicillin delabelling, such as longer hospital stays, and higher mortality rates, were greater than we would expect from the aforementioned studies. We found that this knowledge gap should be closed by education and increasing this knowledge might contribute to motivate clinicians to perform PAD and aid PAD implementation in the WNHR.

For nurses participating in our study, the prospect of a simplified PAD procedure was deeply motivational, and this source of motivation has not been reported in earlier studies. This may be related to the fact that several nurses in our study had performed skin testing and administered intravenous antibiotic test doses previously, and therefore noticed the possible benefits of a simplified procedure. This may also be the reason why the nurses were more concerned about optimising the organisational context and preparing for a possible adverse reaction, rather than the physicians’ anxiety about causing an adverse reaction. Also, the risk and responsibilities of patient monitoring post-challenge when performing PAD remains unchanged for the nurses. As mentioned above, the Norwegian health system has a rather low degree of hierarchy, and nurses work with a large degree of autonomy. However, the Norwegian health system still has clear definitions of responsibility concerning clinical decisions, the decision to perform PAD lays with the physician, and not the nurse, explaining why they mostly stated, “they will do what the doctor decides” whereas the physicians reported a justified fear of consequences if their patients suffer negative outcomes induced by PAD.

The informants demonstrated a motivation for further education and training in PAD. They used the interview to ask several questions to the interviewer concerning PAD, as they knew the interviewer works with drug allergies on a daily basis. Clinicians volunteering to participate in interviews have already demonstrated a willingness to participate, and participation has shown to increase learning [36]. Hence, this motivation to learn might not be present in all clinicians expected to perform PAD. If the same motivation to learn could be mobilized in other clinicians, this would give a starting point for safe and sustainable PAD implementation.

The informants reported a sense of shame towards their current clinical procedures in PAD, as they were outdated and rarely performed. This demonstrates a tension for change [37, 38]. Tension for change has earlier been recognized as a necessary antecedent for successful implementation, highlighting the need for change should be utilised to aid uptake and motivate clinicians to perform PAD on an everyday basis [39].

### Organisational structures

Clinicians make a vast number of decisions regarding patients every day and have been reported to manage this by applying “fast and frugal heuristics” [40, 41]. Hence, new methods must prove both their evidence base and fit rapid decision patterns to be accepted into practice. The informants readily admitted that this way of thinking and working was part of the reason why they did not perform PAD. Alternative antibiotics could easily be prescribed, and this was the established way of handling patients declaring a penicillin allergy. Raising the bar for prescribing alternative antibiotics has been called for in other studies [9, 35], but the practicalities of doing so are still unexplored. Our informants suggested amending the electronic patient journals to nudge them away from prescribing other antibiotics. Additionally, they suggested amending the Norwegian antibiotics guidelines, so when a penicillin is the advised treatment, the guideline should recommend performing PAD, in addition to the suggestion of an alternative antibiotic.

The informants also mentioned that they are trained to respect drug allergy labels. Changing established truths and practices are described as de-implementing and unlearning. To de-implement is a part of implementation, as two sides of a mirror, since the uptake of new methods depends on de-implementing old ones [42, 43]. To implement PAD further, trust in the new methods must be endorsed and at the same time old guidelines- and clinician reaction patterns de-implemented. On a longer timescale health care students and providers will need updated education concerning drug allergy warnings for PAD programs to be sustainable.

The informants stated that preformed logistics, where PAD is readily available in the workflow, including preformatted text in the electronic patients’ charts were desirable. Organisational structures are the sum of how procedures are performed, by whom, and how decisions are made to obtain an organisational aim [44]. As the organisational workflows of hospitals and clinicians differ, there was a call for both paper forms, computer forms and mobile phone apps, mirroring the complexity of health care and indicating that one size will not fit all. The informants emphasised that a PAD not embedded in their workflow would seldom be performed, as it would not be prioritised over other tasks at hand. The less disruptive and more bundled into current care the methods are, the more likely one is to achieve sustainable implementation of PAD, which is in line with earlier findings [9, 10, 26].

### Strengths and limitations

Interviewing both nurses and physicians enabled us to obtain a broader view of the topic. Having multidisciplinary teams both creating the study and analysing the results also ensured different perspectives on our research questions (nurse, pharmacist, and doctors with experience from occupational medicine, infection medicine and pulmonology), in addition to the participation of both male and female researchers. Recruiting informants from multiple kinds of hospitals (local, regional and university hospitals), adds transferability to our findings. Earlier studies from Great Britain [9] and the United States of America [10] were single-centred, and we believe that information from different types of hospitals increases the validity of our study. Furthermore, the informants worked across several types of departments, their experience in PAD varied from none to experienced, and their professional seniority ranged from newly educated to experienced clinicians. We performed interviews with groups consisting of nurses and physicians in as equal numbers as possible, but also performed interviews with physicians and nurses on their own, to ensure that the power dynamics between professions did not restrict the informants’ answers and ensure internal validity.

All authors work at WNHR hospital (six physicians, one nurse and one pharmacist) and have a thorough understanding of the everyday working order of the service. We were aware of the high hierarchical position we occupy as senior clinicians, and that this, together with our employment in the same health region as the informants, might have influenced the answers. However, as the informants spoke openly about their own vulnerability and professional anxiety, we assess the internal validity to be high.

This study has several limitations. First, the informants all work in one Norwegian health region, potentially reducing the transferability to other countries and different health care systems. Nevertheless, as the study included all types of public hospitals in Norway, the results are most likely transferable to other Norwegian health regions, and other Western countries with similar healthcare organisations and penicillin prescription practices. Second, none of the informants worked in a surgical department at the time of interview. This might limit the transferability to surgical departments, although the informants’ answers were in line with results from previous studies in other countries [9, 10]. Also, all informants were nominated by their local leader creating a possible bias, as leaders could be inclined to nominate the most skilled clinicians. The study did not explore the patients’ perspective in PAD. Earlier studies have reported that patients can be reluctant to undergo PAD [45, 46]. Few of our informants had performed PAD regularly, but none of the informants reported patients opposing to PAD. This might change when implementing the PAD on a larger scale and must then be addressed.

## Conclusion

Clinicians need psychological safety to perform PAD. Many of the other obstacles for PAD reported earlier, such as time constraints and practical organisation, were deemed manageable by our informants if they felt psychological safe. The informants voiced a need for empowerment through education, and through leadership-, collegial- and guideline support to experience psychologically safe performing PAD. The need for psychological safety was reported more profoundly than in earlier studies, probably due to social factors.

PAD should be a part of everyday practice in a contextually adapted logistic chain. In addition, the clinician’s high level of motivation towards providing the best health care possible should be utilised to aid sustainable PAD implementation. The knowledge gained from this study will aid sustainable implementation of PAD in Norway.

### Electronic supplementary material

Below is the link to the electronic supplementary material.

Supplementary Material 1

Supplementary Material 2

Supplementary Material 3

Supplementary Material 4

## Data Availability

The datasets are available from the corresponding author upon reasonable request. General public availability is not guaranteed for confidentiality reasons.

## **Abbreviations**

WNHR: Western Norway Health Region
PAD: Penicillin allergy delabelling
MBA: Marie Bjørbak Alnæs
BS: Brita Skodvin
MAS: Margrethe Aase Schaufel
GVK: Grete Velure Kalleklev
JAJ: Jan Anker Jahnsen
