# Supplementary material for: “What if the patient has a severe reaction, and it is my fault?” A qualitative study exploring factors for sustainable implementation of penicillin allergy delabelling
Source: Antimicrob Resist Infect Control. 2024 Sep 2;13:97. doi: 10.1186/s13756-024-01456-8 (PMC11368001; doi:10.1186/s13756-024-01456-8)
Supplement: Supplementary file 1 — Supplementary Material 1 [file 13756_2024_1456_MOESM1_ESM.pdf]

Semistructured Interview guide:

Introduction
Welcome, thank you for participating! My name is Marie Bjørbak Alnæs. I am a physician and at the moment I am also researching declared penicillin allergy. I am going to interview you about your thoughts about what could facilitate, and what are barriers for implementing a method to delabel penicillin allergy on the lowest possible level.

It is known that the label ”penicillin-allergic” in the patients charts or declared by the patient often is not correct. This impacts the health care of the patient. We are going to have a conversation about how you treat patients with declared penicillin allergy and how a tool for exploring declared penicillin allergy should be applicable in everyday practice. I want your thoughts and suggestions about how this work could be easier and better. The results from the interviews are to be published as part of my research in my PhD project and all data will be anonymized and confidential. You can pull back from the study at any time, and until the data has been published. I expect the interviews to take about an hour. Any questions? If not, please sign the consent forms bevor we start.

We start with a small presentation round. If you could tell me your first name, what profession you have, and your experience concerning penicillin allergy delabeling?

Culture
Now we shall talk about how declared penicillin allergy is managed in your hospitals and departments today.
1. Do you remember the last patient you had with declared penicillin allergy that had an indication for being treated with penicillin? How did you manage this patient?

Tools and guidelines
2. What tools do you use for managing patients with declared penicillin allergy?
3. Do you know any guidelines for managing patients with declared penicillin allergy? What do you think about these?
4. What tools would you like to have to evaluate the patients declared penicillin allergy more easily? Do you for instance prefer electronic support in the patients’ charts, forms on paper, electronic tools such as apps and web pages, or something different?

Structural support and education
5. Are there any issues concerning declared penicillin allergy in a patient you would contact others for advice about?
6. How did you learn about penicillin allergy?
7. Is there any education about penicillin allergy you have missed?

Feelings
8. What can be hard considering patients with declared penicillin allergy?
9. What do you think about examining the patient for delabeling of the declared penicillin allergy?
10. What would you need to feel safe to perform penicillin allergy delabeling?
11. What would motivate you to perform penicillin allergy delabeling?

Use of tools
In many countries there are questionnaires used for examining the declared penicillin allergy as a support towards evaluation of the risk of true penicillin allergy, and a risk stratification before a possible drug provocation test.

Følsomhet Intern (gul)

12. Do you have any experience with such questionnaires and what do you think about these procedures?

As some of you know my research group have developed a Norwegian clinical pathway for delabeling penicillin allergy. A paper version of the forms in the pathway has been presented to you all.

13. What do you think about these forms?
How can they be improved to fit your everyday clinical practice?
In what form would you like the tools to be available, to be applicable and useful?

Own organization
If your hospital was to develop and implement a program to help you as clinicians to evaluate and delabel declared penicillin allergy;
14. What elements does such a program need to for it to be useful for you?
15. What do think is needed for such a program to succeed? And what can be hard when implementing such a program?

Leadership
16. How do you think the suggestion of a penicillin allergy delabeling program would be met by the leadership in your department and hospital? What is needed for your leadership to join in on this?

Ending
Thank you for the conversation, I will summarize what information I have gained from our conversation. Do you agree or not?
Is there anything else you would like to add? If not, thank you so much for participating! All the responses will be used for aiding implementation of declared penicillin allergy in Norway.

Følsomhet Intern (gul)
