# Supplementary material for: “What if the patient has a severe reaction, and it is my fault?” A qualitative study exploring factors for sustainable implementation of penicillin allergy delabelling
Source: Antimicrob Resist Infect Control. 2024 Sep 2;13:97. doi: 10.1186/s13756-024-01456-8 (PMC11368001; doi:10.1186/s13756-024-01456-8)
Supplement: Supplementary file 3 — Supplementary Material 3 [file 13756_2024_1456_MOESM3_ESM.docx]

*Overview of the Systematic Text Condensation Analyzing Process in the Study*

| **STEP 1:**  **Getting a total impression** | **STEP 2:  Identifying meaning units** | | **STEP 3:  Abstracting the contents of individual meaning units** | **STEP 4:  Summarizing the findings** |
| --- | --- | --- | --- | --- |
| *Process:* a) The authors read the transcribed interviews separately b) Discussed the total impression to consensus | *Process:*  a) The authors coded the data separately  b) Discussed the codes to consensus within the codes | | *Process:* a) The authors analyzed the contents  b) Several discussions to consensus | *Process:*  b) The authors discussed the findings against the transcribed interviews  c) The authors found direct statements to elucidate units of meaning and discussed to consensus |
| ***Identified total impression: themes*** | ***Identified meaning units: codes*** | | ***Abstracted contents: code groups*** | ***Summarizing: categories*** |
| **A want for empowerment:** -knowledge -Tools/guidelines -building knowledge and competence -Feel safe and not afraid of making errors / inflicting harm on patient -unlearning outdated and wrong knowledge. -Surprisingly large level of anxiety towards PAD.  -Clinicians crave knowledge about PAD.  **A want for good everyday logistics** Everything must be automatized/ without hassle to be done. PAD must be -Practical -Simple -readily available -Time is always short, the benefit of spending time on PAD must be clear. -Clinicians worry that PAD could be a hassle.  **Lighthouses** Resourceful colleagues that can be approached when questions arise. Facilitators and facilitating it to be easy to do PAD Someone to keep the motivation for PAD up. The main motivation: Better patient care. Pave the way for sustainable change.  Want leadership  Want clinical consensus in PAD.  **motivation** useful for the patient Useful for the department and health care system Useful globally | ***Code*** | ***Themes sorting under each code*** | **A need for psychological safety** -reduce the anxiety towards possible negative consequences of PAD; for patient and clinician -Experience support from leadership and colleagues -Need for empowerment trough increased knowledge and experience -Guideline support.  **Utilize clinicians’ inherent motivation** -provide best possible care for patient -Contribute to community (increased health, less antibiotic resistance, less costly treatment) -The prospect of simplified procedures | 1. **A need for psychological safety** **-** *Clinicians’ anxiety when performing PAD must be addressed and decreased*   *- Clinicians need empowerment and support to perform PAD*   1. **Utilize clinicians’ inherent motivation** **-** *Clinicians want to provide the best possible healthcare* *- Clinicians want to reduce antimicrobial resistance and negative impacts of penicillin allergy labeling* *- Clinicians are motivated by the prospect of a simplified clinical procedure:* 2. **Optimal organizational structures** **-** *Clinicians need a seamless workflow adapted to their working context* *- Clinicians need appointed clinicians to lead implementation* |
|  | empowerment | Knowledge Tools Guidelines Increase Competence Education  Training |  |  |
|  | logistics | The PAD method must be: -Practical -easy -Available  -Integrated in everyday logistics  -Multimodal approach (some wants an app others want paper) | **Optimal organizational structures** -Seamless workflow (adaptable to local context) -Lighthouses to take care of team and aid cooperation across professions.  Facilitate clinicians’ inherent motivation: For providing best possible health care  -contribute to societal benefit  - being able to use simpler procedures |  |
|  | Lighthouses | A person/team who: -lead implementation. -aid cooperation across professions -lead education and training.  -Someone available for questions and help |  |  |
|  | motivation | -Give best patient care -Have updated knowledge and methods -To use simplified methods -The gains of PAD for patient and society.  -The gains of PD for health care system  -Getting the group form thinking of PAD to performing PAD |  |  |
|  | Uncertainty | -Afraid of negative patient outcomes.  -Afraid of new methods that go against old knowledge.  -Afraid of negative consequences for them as physicians, from colleagues, leaders and health authorities.  -Uncertain of how, when and on which patients PAD can be performed  -Uncertain responsibilities in PAD performance. |  |  |
|  | Guidelines | -Clear instructions about the when, what, who and how of performing PAD.  -Must exist on hospital and national level.  -Should explain the same method in all guidelines.  -Clinicians report that the existence of guidelines empowers them, and it is a goal to be adhering to guidelines.  -Guidelines make them feel safe to perform PAD. |  |  |
|  | Tools | -Simple  -Must be intuitive and remove doubt of how to further treat the patient  -Must be validated |  |  |
|  | Emotions | -Need to feel safe -Fear -Need for collegial and leadership  -support -Feel the benefit |  |  |
|  | Knowledge | -Know about procedure -Know about possible gains. -Unlearn old wrong knowledge. -The shared knowledge in the department.  -Need to be educated.  -Fill the knowledge vacuum concerning  PAD.  -Experience success in performing PAD and positive outcomes. |  |  |
|  | Leadership | -Know that PAD is supported by leadership.  -Need for clinical leadership and someone to aid implementation. |  |  |
|  | Time | -PAD must take as little time as possible and or demonstrate that time spent on PAD is well invested. -Time restraints are a large barrier for performing PAD. |  |  |
|  | Resources | -Resources are limited overall.  -Need tailored resources for PAD and to see that resources spent on PAD are justified.  -The resources needed must be allocated.  -Clinicians want to know that their resources spent on PAD could save other resources.  -Resources are human, material and time needed to perform PAD |  |  |
