# Supplementary material for: “What if the patient has a severe reaction, and it is my fault?” A qualitative study exploring factors for sustainable implementation of penicillin allergy delabelling
Source: Antimicrob Resist Infect Control. 2024 Sep 2;13:97. doi: 10.1186/s13756-024-01456-8 (PMC11368001; doi:10.1186/s13756-024-01456-8)
Supplement: Supplementary file 4 — Supplementary Material 4 [file 13756_2024_1456_MOESM4_ESM.docx]

**Supplement 2**

Table 2, distribution of informants in the focus groups

| **Interview number (total 8)** | **Number of informants (total 25)** | **Particularities** |
| --- | --- | --- |
| 1 | 1 | Two clinicians could not participate due to acute clinical staff changes. |
| 2 | 4 |  |
| 3 | 5 |  |
| 4 | 3 | Only physician informants |
| 5 | 5 |  |
| 7 | 4 | Only nurse informants |
| 8 | 3 | Online interview using “Teams” (Microsoft) due to organisational challenges. |

*Table 2: Distribution of informants in the focus groups*
